# Exposure measurement error when assessing current glucocorticoid use using UK primary care electronic prescription data

**DOI:** 10.1002/pds.4649

**Published:** 2018-09-28

**Authors:** Rebecca M. Joseph, Tjeerd P. van Staa, Mark Lunt, Michal Abrahamowicz, William G. Dixon

**Affiliations:** ^1^ Arthritis Research UK Centre for Epidemiology, Centre for Musculoskeletal Research, School of Biological Sciences, Manchester Academic Health Science Centre The University of Manchester Manchester UK; ^2^ Health eResearch Centre, Centre for Health Informatics, School of Health Sciences, Manchester Academic Health Science Centre The University of Manchester Manchester UK; ^3^ Faculty of Science, Division of Pharmacoepidemiology and Clinical Pharmacology Utrecht University Utrecht The Netherlands; ^4^ Department of Epidemiology, Biostatistics & Occupational Health McGill University Montreal Canada; ^5^ NIHR Manchester Biomedical Research Centre Central Manchester University Hospitals NHS Foundation Trust, Manchester Academic Health Science Centre Manchester UK; ^6^ Rheumatology Department Salford Royal NHS Foundation Trust Salford UK

**Keywords:** electronic health records, glucocorticoids, pharmacoepidemiology, validity (epidemiology)

## Abstract

**Purpose:**

To quantify misclassification in glucocorticoid (GC) exposure defined using UK primary care prescription data.

**Methods:**

A cross‐sectional study including patients with rheumatoid arthritis prescribed oral GCs in the past 2 years. Glucocorticoid exposure based on electronic prescription records was compared with participant‐reported GC use captured using a paper diary. Prescription data (containing information about prescriptions issued but no dispensing information) was provided by the Clinical Practice Research Datalink. The following variables were defined: current use and dose of oral GCs and if (and when) participants had received a GC injection. For oral GCs, self‐reported use was taken to represent “true” exposure. A dataset representing a hypothetical population was generated to assess the impact of the misclassification found for current use.

**Results:**

A total of 67 of 78 study participants (86%) were correctly classified as currently on/off oral GCs; 32/38 (84.2%) participants reporting current GC use and 35/40 (87.5%) participants not reporting current use were correctly classified. Estimated values of current dose were imprecise (correlation coefficient 0.46). Concordance between reported and prescribed GC injections was poor (kappa statistic 0.14). Misclassification bias was demonstrated in the hypothetical population: For “true” relative risks of 1.5, 4, and 9, the “observed” relative risks were 1.33, 2.48, and 3.58, respectively.

**Conclusions:**

Misclassification of current use of oral GCs was low but sufficient to lead to significant bias. Researchers should take care to assess the likely impact of exposure misclassification on their analyses.

**KEY POINTS**

- Glucocorticoid exposure defined using UK electronic prescription data (prescriptions issued) was compared with participant self‐report, taking self‐report to represent true exposure.
- Current use of oral glucocorticoids was correctly classified for 86% of participants, with a false positive rate of 6.4% and false negative rate of 7.7%.
- Estimates of daily dose were not significantly biased but were imprecise, with a mean (SD) absolute difference between estimated and reported dose of 3.2 (4.2) mg (prednisolone equivalent).
- Concordance between prescribed and reported glucocorticoid injections was low (κ = 0.14) indicating primary care prescription data alone are not a reliable source of information about glucocorticoid injections.

## 1 INTRODUCTION

Databases of UK primary care electronic health records (EHR), such as the Clinical Practice Research Datalink (CPRD), are commonly used for pharmacoepidemiological research, including drug safety or effectiveness studies.^1^ Such studies aim to estimate the association between exposure to a particular medication and a beneficial or adverse outcome. The accuracy of such an estimate is affected by how accurately the variables of interest are measured.

In a drug safety or effectiveness study, the underlying exposure of interest is actual intake of the drug by patients.^2^ For studies set within UK primary care EHR, drug exposure is typically defined using information about prescriptions issued to patients. Estimates of drug exposure based on primary‐care prescriptions are only a proxy measure of true drug exposure, and there are numerous reasons why such estimates may differ from actual drug intake. Such datasets do not capture medication accessed outside of primary care, including drugs issued in a hospital setting or over the counter.^3^ In addition, prescription data do not reveal whether the patient filled that prescription or took the medication as prescribed.

Differences between true and estimated drug exposure (known as exposure measurement error or misclassification) can lead to misclassification bias, potentially masking the true association between the drug and outcome of interest.^2^ While there have been numerous studies validating diagnoses in UK primary care research databases,^4^ the accuracy of measurements of drug exposure have been largely unexplored. Similarly, most pharmacoepidemiology studies recognise the potential for exposure misclassification,^5^, ^6^ but few attempt to quantify the extent or impact of the resulting errors^7^, ^8^ or correct for such errors.^9^

The purpose of this study was to quantify errors in the measurement of glucocorticoid (GC) exposure based on UK primary care prescription data. Glucocorticoids are an interesting example due to multiple and sometimes complex prescribing patterns and the possibility of secondary care prescribing. In addition, GCs are associated with a range of side effects,^10^ which may lead to nonadherence to GC regimes by patients.^11^, ^12^

Exposure to GCs estimated using primary care prescription data was compared with patient‐reported GC use captured using diaries. The objective was to quantify misclassification in estimations of current use of oral GCs. Misclassification in daily dose and in GC injections was also explored. The impact and importance of this misclassification was demonstrated using a generated dataset representing a hypothetical population with levels of exposure misclassification informed by the results of the main study.

## 2 METHODS

### 2.1 Design and setting

This analysis was conducted as part of a study investigating GC use and adrenal insufficiency in patients with rheumatoid arthritis (RA) (the “SAIRA” study).^13^This was a cross‐sectional study with data collection nested within UK primary care EHR. Participants were recruited through general practices across England. Between October 2015 and April 2016, participants collected a saliva sample and completed a diary providing information about their current and past use of GCs. The present analysis makes use of the information collected in the diaries. This information was combined with EHR data provided by the CPRD.^14^

The study was approved by the NHS National Research Ethics Service Committee London‐Bromley (reference 14/LO/1335) and the Independent Scientific Advisory Committee (reference 14_145RA).

### 2.2 Clinical Practice Research Datalink

Clinical Practice Research Datalink is a database of pseudonymised UK primary care electronic health records. General practices contribute information prospectively to CPRD. Information captured includes demographic characteristics, symptoms and diagnoses, and all prescriptions issued by the general practices. Clinical Practice Research Datalink provided the complete, depersonalised, EHR for the participants in this study, as of June 2016. The design of the study precluded linkage of CPRD data to identifiable patient data, except by the relevant general practice.^13^

### 2.3 Participants

For the SAIRA study,^13^ eligible participants were patients with RA (identified within CPRD using a validated algorithm^15^), over the age of 16, registered at an English general practice, prescribed oral GCs within 2 years of the study, and had no record of any conditions or medications known to affect adrenal function. All participants had at least 2 years of follow‐up within CPRD. Potentially eligible patients were screened for suitability by their GP. General practices mailed invitations to potential participants. Participants were recruited by the study team at the University of Manchester and provided written consent to take part.

### 2.4 Self‐reported GC use

Paper‐based diaries were used to collect information about GC use from participants (see Supplementary S1). Participants completed the diaries at home on a single occasion, shortly after waking and before taking any GCs. An illustrated list of possible GC tablets was provided to aid participants in completing the diary. Where questions about GC use were left blank this was interpreted as no use. The following variables were defined using the diaries: exposed/not exposed to oral GCs in the past 24 hours; total dose of oral GCs (mg, prednisolone equivalent dose) taken in the past 24 hours; ever given a GC injection; and number of months since last GC injection.

### 2.5 Prescribed GC use

Prescriptions for oral and injected GCs were identified within the EHR provided by CPRD. The available data represent written prescriptions rather than filled prescriptions. A record is generated for each prescription written, including each prescription refill. Code lists for oral and injected GCs were generated by identifying all products corresponding to the British National Formulary^16^ chapter 6.3.2 (“Glucocorticoid therapy”) within the product dictionary provided by CPRD and by searching the dictionary using a prespecified list of drug substances. To determine whether a patient had an active prescription on a particular date, it was necessary to define the start and end of each prescription. For this, we used an algorithm^17^ that takes into account multiple sources of duration information within CPRD and accounts for overlapping prescriptions (Supplementary S2). The daily dose of GCs was calculated as the strength multiplied by the number of tablets per day. Doses were converted to prednisolone‐equivalent dose.

### 2.6 Participant characteristics

The following demographic characteristics were defined using the CPRD data: gender, age, and socio‐economic status (SES) defined using the Townsend score.^18^ Socio‐economic status information is provided as a linked dataset by CPRD and is missing for general practices that have not agreed to the linkage (approximately 45% of CPRD practices). Duration of RA was calculated from the date the participants first met the criteria for RA according to the algorithm.^15^

### 2.7 Analysis

Characteristics of the study population on the diary date were summarised using proportions or the median and interquartile range (IQR).

Misclassification in oral GC use (currently/not currently exposed) was quantified by comparing self‐reported GC with the status determined from the prescription data. Self‐reported use was taken to be the true exposure. The sensitivity, specificity, positive predictive value (PPV), and negative predictive value (NPV) were determined. Exact (binomial) confidence intervals (CIs) were calculated. Difference tests (Kruskal‐Wallis and chi‐squared tests) were used to investigate possible associations between participant characteristics and misclassification of current GC use.

Daily dose according to self‐report and the prescription data were summarised. Scaled normal density plots were drawn to visualise the distributions of the two measures of daily dose. Measurement error in continuous variables consists of a systematic error (bias) and subject error (precision).^2^ Taking self‐reported dose to be true exposure, bias was assessed by the difference between the means of the two measurements, with 95% CIs. The Spearman rank correlation coefficient was calculated to estimate concordance between the dose estimated from prescription data and the “true” reported dose. The mean (standard deviation, SD) absolute difference between the two measurements was calculated as a measure of precision. Results were calculated for all participants and then including only participants correctly classified as current users.

Finally, whether participants ever received a GC injection according to self‐report or the prescription data were compared, using Cohen kappa statistic to measure concordance (neither measurement was judged to be the “true” exposure). The timings of the most recent GC injection according to both measures were summarised.

To demonstrate the potential impact of exposure misclassification at the population level, a dataset representing a hypothetical population was generated using Stata. A dataset of 100 000 observations was generated and “true” exposure (binary) was set to 50%. “Observed” exposure was then generated, conditional upon the true exposure and the sensitivity and specificity found in the main study. Nondifferential misclassification was assumed. An outcome variable was then generated conditional upon the true exposure and independent of the observed exposure. The relative risk (RR) for observed exposure was calculated (details in Supplementary S3).

All data cleaning and analyses were performed using Stata/MP 13.1 (StataCorp LP, Texas).

## 3 RESULTS

For the SAIRA study, invitations were sent to 526 patients, 117 participants were recruited, and 86 participants returned their diaries (the full flow of participants has been described previously^13^). All of these 86 participants are included in the present analysis. The numbers and characteristics of patients at various stages of recruitment are shown in Supplementary S4: Participants were similar to the whole eligible population in terms of age and gender but tended to be of a higher SES. One participant did not provide a diary completion date, and a further seven were excluded as the last data uploaded by their practice preceded the diary completion date. Of the remaining 78 participants, 72% of participants were female, the median age was 68 years (range, 28‐89), and the median duration of RA was 7.7 years (Table 1).

**Table 1:** Participant characteristics on index date according to classification of current use of oral GCs^a^

	All	Correctly Classified	Misclassified	Difference Test
Number of participants	78	67	11	…
Female, n (%)	56 (71.8)	48 (71.6)	8 (72.7)	χ^2^ (1)=0.01, *P* = 0.941
Age, median (IQR)	68 (60‐75)	68 (61‐74)	61 (49‐76)	KW (1)=0.90, *P* = 0.343
Townsend score quintile, median (IQR) (1 = least deprived)	2 (2‐3)	2 (2‐3)	2.5 (1.5‐3.5)	χ^2^ (4)=0.73, *P* = 0.948
RA duration (years), median (IQR)	7.7 (2.8‐10.4)	7.8 (3.6‐10.4)	5.3 (1.9‐20)	KW (1)=0.21, *P* = 0.651
Cumulative oral GC dose (g)^b^, median (IQR)	2 (0‐4)	1.8 (.4‐4.4)	2.3 (1.3‐3.3)	KW (1)=0.11, *P* = 0.736
Percentage of time on oral GCs^b^, median (IQR)	45% (8‐93%)	45% (793%)	50% (9‐95%)	KW (1)=0.09, *P* = 0.769

Abbreviations: GC, glucocorticoid; IQR, interquartile range; KW, Kruskal‐Wallis test; RA, rheumatoid arthritis.

a Townsend score was missing for nine participants.

b In the previous 2 years, according to prescription data.

### 3.1 Oral GCs: Current use

**Figure 1:**
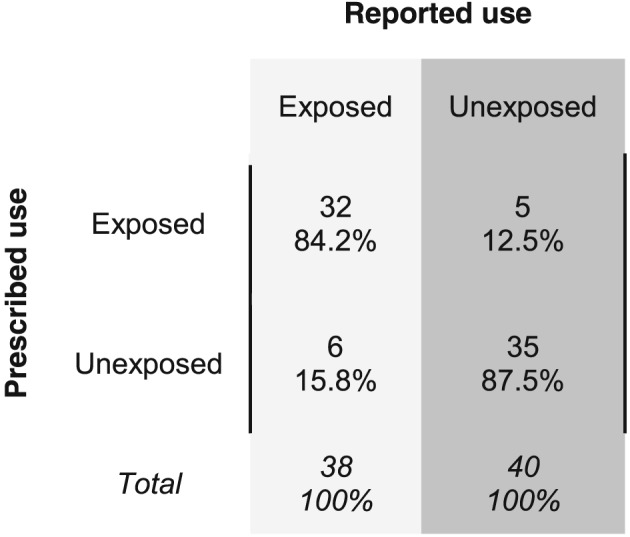
Misclassification matrix for current use of oral glucocorticoids. Current use (exposed vs unexposed) according to prescription data is compared with current use according to patient self‐report. The figure shows the percentages of patients in each category, according to self‐report, who were correctly classified or misclassified in the prescription data.

Out of 78 participants, 38 (49%) reported currently taking oral GCs on the diary completion date while 37 (47%) had an active prescription for oral GCs. Using the prescription data, 32 of the 38 participants reporting current use and 35 of the 40 participants not reporting current use were correctly classified (Figure 1). Therefore, using the prescription data to predict patient‐reported current GC use the sensitivity was 84.2% (95% CI, 68.7‐94.0%) and the specificity was 87.5% (95% CI, 73.2‐95.8%) (Figure 1). The PPV was 86.5% (71.2‐95.5%), and the NPV was 85.4% (70.8‐94.4%). Overall, the current GC exposure statuses of 67 (86%) participants were correctly classified in the prescription data compared with self‐report. There were no obvious differences in participant characteristics between those correctly classified and misclassified (Table 1).

### 3.2 Oral GCs: Current dose

The estimated current daily dose matched the reported dose for 42 of the 78 participants; this includes the 35 participants who were correctly classified as noncurrent users (hence with a daily dose of 0 mg). The estimated dose was greater than the reported dose for 20 participants and less than the reported dose for 16 participants. The mean reported and prescribed daily doses are presented in Table 2. Overall, the bias (mean difference) was 0.2 mg (95% CI, −1.0 to 1.4 mg), the mean (SD) absolute difference between the prescribed and reported dose was 2.4 (4.7) mg, and the Spearman correlation coefficient was 0.70 (*P* < 0.001).

**Table 2:** Mean dose of oral GCs according to self‐report and prescription data^a^

	All Participants (n = 78)	Current GC Users (n = 32)
Mean (SD) reported dose, mg	3.4 (4.9)	6.6 (3.2)
Mean (SD) prescribed dose, mg	3.6 (5.3)	7.6 (5.8)
Mean (SD) absolute difference, mg	2.4 (4.7)	3.2 (4.2)

a Values are shown for all participants (n = 78) and for those who were correctly classified as current GC users (n = 32). GC, glucocorticoid; SD, standard deviation.

**Figure 2:**
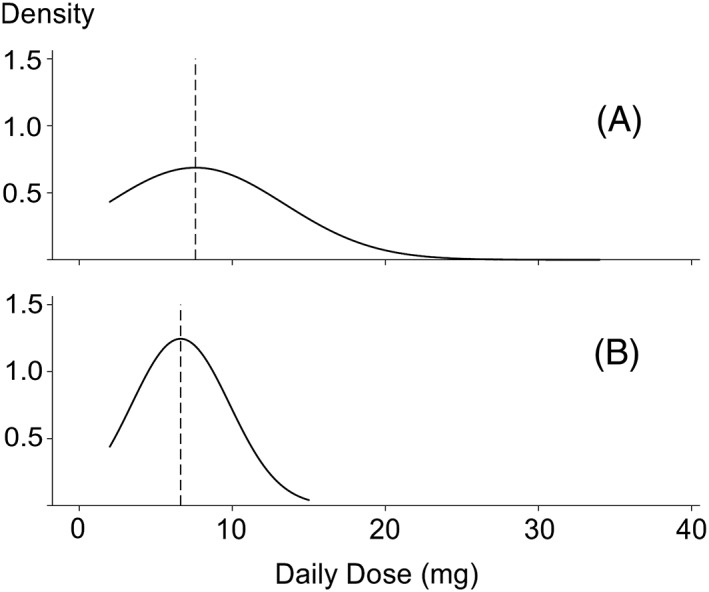
Scaled normal density plots for daily dose of oral glucocorticoids according to prescription data (A) and self‐report (B). The dashed vertical lines are the sample means.

Considering only the 32 participants correctly classified as current oral GC users, the estimated daily dose matched the reported daily dose for seven participants, was an overestimate for 15 participants, and was an underestimate for 10 participants. For this subset of participants, the bias was 1.0 mg (95% CI, −0.9 to 2.9 mg). The mean (SD) absolute difference in dose values was 3.2 (4.2) mg, and the Spearman correlation coefficient was 0.46 (*P* = 0.009). The distributions of the two measures of daily dose for these 32 participants are shown in Figure 2; this figure highlights the small difference in the means but larger variance in the daily dose estimated from prescription data.

### 3.3 GC injections

**Figure 3:**
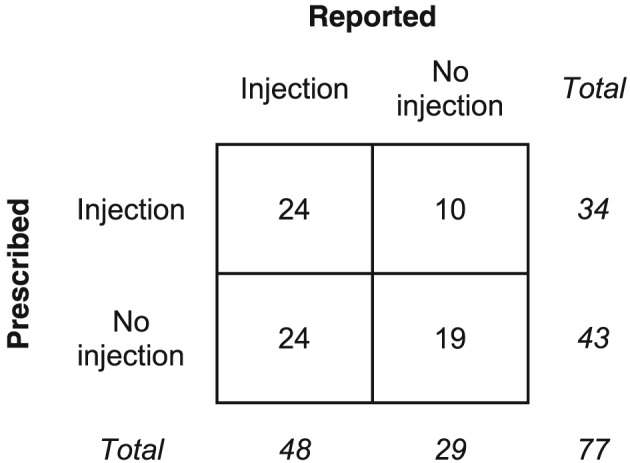
Self‐reported vs prescribed GC injections. Cross‐tabulation of ever receiving a GC (glucocorticoid) injection according to self‐report or primary care prescription records.

Excluding one participant who reported an injection but was unsure whether it was GC, 48 of 77 (62%) reported ever having a GC injection and 34 (44%) had a GC injection in their primary care prescription records. Of the 48 participants reporting a GC injection, 24 (50%) had no GC injection in their prescription records, while 10 (34%) of the 29 participants who did not report a GC injection did have a GC injection in their prescription records (Figure 3). The kappa statistic for concordance between the datasets was 0.14 (95% CI, −0.06 to 0.34), indicating little to no agreement. Reported injections were on average more recent than prescribed injections, a median of 0.7 (range 0‐8.5) years ago compared with 2.7 (range 0‐24) years ago.

### 3.4 Impact of misclassification

The sensitivity and specificity found for current use of oral GCs (84.2% and 87.5%, respectively) were applied to the hypothetical population generated using Stata. In this dataset, while the “true” exposure rate was set to 50%, the “observed” exposure rate was 48.4%. The RR of the outcome according to the true exposure classification was varied. For true RRs of 1.5, 4, and 9, the observed RRs were 1.33, 2.48, and 3.58, respectively.

## 4 DISCUSSION

This study aimed to quantify errors in the measurement of exposure to oral GCs based on UK primary care prescription data. Agreement between self‐reported use of oral GCs and use estimated from prescription data was high: 86% of participants were correctly classified as current or noncurrent users using the prescription data. However, the hypothetical study demonstrated that this level of misclassification can lead to important bias. A real study would also involve sampling variation around the population RR.

On average, estimates of current daily dose were not significantly biased. For current GC users, the estimates of dose were on average 1 mg (prednisolone equivalent) higher than the reported doses. However, agreement between the two measurements was fairly low (Spearman correlation coefficient 0.46), and the mean (SD) absolute difference was 3.2 (4.2) mg. This measurement error could introduce bias in analyses, as even low levels of imprecision can lead to bias towards the null (eg, page 80 in White 2005^2^).

Concordance between self‐reported and prescribed GC injections was very low, with a kappa statistic of 0.14 and confidence intervals crossing 0. In this case, neither measurement was considered to be more accurate than the other. Patients' recall of historical injections is unlikely to be accurate, while injections given in secondary care will not be captured in the primary care prescription records.

Previous studies have predominately validated pharmacy dispensing data rather than prescription data. These studies have reported levels of misclassification varying according to the class of medication.^19^, ^20^, ^21^ One previous study attempted to validate prescriptions of low‐dose aspirin using data from The Health Improvement Network (THIN), which, like CPRD, captures UK primary care data^22^; this study collected additional information from general practices in order to estimate misclassification due to over‐the‐counter prescribing of aspirin. To our knowledge, the current study is the first CPRD study to attempt to validate prescription‐based estimates of drug exposure.

There are several reasons why GC exposure estimated using primary care prescription records may differ from true drug exposure. First, primary care records may not capture all medications to which patients have been exposed. Glucocorticoids prescribed in other health care settings, such as rheumatology clinics, are not typically captured in primary care EHR.^3^ Second, receiving a prescription does not guarantee the patient will take their medications as prescribed. Glucocorticoids are associated with a range of side effects that matter to patients and are likely to affect adherence.^10^, ^12^, ^23^ In addition, studies able to link prescribing and dispensing data have demonstrated that a high proportion of prescriptions are never filled by patients.^24^, ^25^ Third, studies set within EHR are reusing data originally collected for purposes other than research. Preparing such datasets for research typically involves making a number of assumptions about the data. The current study used an algorithm to estimate the duration of individual prescriptions based on any duration information provided by the GP. These data processing steps can influence the rate of misclassification in the final dataset. van Staa et al^26^ demonstrated how decisions about exposure duration can impact the degree of misclassification. This has subsequently been demonstrated in a number of observational studies.^27^, ^28^, ^29^, ^30^

There is little literature regarding the definition of dose from prescription data, but it is likely that the measurement error found in the current study was influenced by the quality of the dosage information available in the EHR, patient adherence, and data preparation decisions. The variable used to define dose is derived from free text by CPRD and reflects the average daily dose for a prescription. Complicated dosage regimens (eg, tapering schedules) will not be closely reflected in the final dataset. Potentially, the estimated dose could reflect the average dose taken by patients more closely than it does the dose taken on a particular day. It was not possible to examine this in our data, although a subset of participants (10 of 78) reported a variable dose throughout the week.

In order to quantify misclassification in estimates of true drug exposure based on primary care prescription data, it was necessary to compare estimates to an alternative data source. There are no perfect measures of true drug exposure: only direct observation or measurement of active drug in biological samples can guarantee a drug has been taken, yet these approaches add significantly to the cost and complexity of a study.^2^ Participant self‐report was chosen as a pragmatic source of drug exposure data. There are recognised challenges with self‐reported medication use, and it is possible that the self‐reported data will also differ from true exposure. The diary was designed to minimise the risk of common sources of errors. To limit errors in recalling use of oral GCs, a short recall period (24 h) was used and an illustrated list of the medications of interest was provided. To reduce the likelihood of errors due to social desirability bias,^31^ participants were assured that their responses were confidential. However, there is a residual risk error in the participant‐reported dataset. The study exclusion criteria did not consider factors potentially related to medication self‐report or adherence (eg, cognitive impairment or depressive symptoms), although ability to give informed consent was one of the screening criteria used by GPs. While acknowledging the residual risk of errors, we considered self‐reported of oral GCs to be a more accurate measurement of true drug exposure than prescription‐based estimates. As described above, there are many potential sources of error in prescription‐based estimates, and in the majority of cases, patients have the ultimate responsibility for taking their medications. Self‐reported medication exposure has been used as the reference standard in a number of previous studies validating routinely collected drug exposure data.^20^, ^27^, ^30^ Specifying a reference standard allowed rates of misclassification in estimated GC exposure to be calculated, rather than simply concordance between the datasets. For GC injections, we asked participants to recall injections over a long period, and thus, this data is likely to contain errors; self‐reported injections were therefore not considered to be more reliable than the prescription data.

A limitation of the study is the small sample size and the likelihood of selection bias: as well as influencing the generalisability of the results, it is likely that the estimated rates of misclassification will be biased. The participants in this study may differ in their medication adherence compared with nonparticipants and taking part in the study may have altered the participants' normal drug‐taking behaviour. Overall, we believe the study population are likely to have higher rates of adherence compared with the general population and therefore that the rates of misclassification reported in this study are likely to be an underestimate. Furthermore, as discussed earlier, multiple factors influence the likelihood of misclassification, including the particular drug studied.^19^, ^30^ As the current study focussed on one particular drug class (GCs) and a particular patient population (patients with RA willing to take part in research), these results are not necessarily generalisable beyond this particular setting. Furthermore, this study included patients with established RA taking on average low GC doses: in early RA and other diseases higher GC doses and more complex treatment regimens may be used. However, the results are useful as they exemplify the problem of misclassification. Finally, the estimated levels of measurement error and the hypothetical study rely on the assumption that self‐report is an accurate measure of drug exposure. Any errors in the quantification of misclassification will affect the results of the hypothetical study.

With these caveats in mind, the results of this study suggest that researchers using these datasets need to consider the potential impact of exposure misclassification on their analyses. Techniques exist to account for misclassification in analyses whether or not validation data are available (see Corbin et al^32^). If a quantitative approach is not used to assess bias, results should be presented cautiously without underplaying the risk of misclassification bias.^33^

In conclusion, when using UK primary care prescription data to define exposure to GCs and assuming participant self‐report to represent true exposure, the rate of misclassification of current use of oral GCs was low, but sufficient to lead to important misclassification bias in our hypothetical example. Measurement error was high for both current dose of oral GCs and exposure to GC injections, and primary care prescriptions may not be reliable as the sole data source for GC injections. Researchers using prescription data to define drug exposure should, as a minimum, consider the risk of measurement errors and the likely impact of misclassification bias on their analyses.

## ETHICS STATEMENT

The study was approved by the NHS National Research Ethics Service Committee London‐Bromley (reference 14/LO/1335) and the Independent Scientific Advisory Committee (reference 14_145RA). All participants gave consent to take part in the study.

## CONFLICT OF INTEREST

The authors declare no conflict of interest.

## Supporting information

Data S1: Supporting information

Data S2: Supporting information

Data S3: Supporting information

Data S4: Supporting information
