# Supplementary material for: Exposure measurement error when assessing current glucocorticoid use using UK primary care electronic prescription data
Source: Pharmacoepidemiol Drug Saf. 2018 Sep 28;28(2):179–86. doi: 10.1002/pds.4649 (PMC6492099; doi:10.1002/pds.4649)
Supplement: Supplementary file 1 — Data S1: Supporting information [file PDS-28-179-s001.docx]

Steroid therapy and adrenal insufficiency in patients with rheumatoid arthritis (SAIRA)

*This document is reproduced from: Joseph RM, Ray D, Keevil B, van Staa, T & Dixon, WG. Low salivary cortisol levels in patients with rheumatoid arthritis exposed to oral glucocorticoids: a cross-sectional study set within UK electronic health records. RMD Open (accepted).*

*Available under a creative commons attribution licence (CC BY 4.0* [*http://creativecommons.org/licenses/by/4.0/*](http://creativecommons.org/licenses/by/4.0/)*)*

Participant Diary

To help us make sure we have accurate information about the steroids you have used recently, we would like you to fill in this diary on the day you take your saliva sample. It includes questions about any steroid tablets you have taken recently and steroid injections you have been given.

There are also some questions about whether you sometimes miss a dose of your steroid tablets or take more than you have been told to. Please be assured that any information you provide in this diary will not be shared with your doctor or any other healthcare provider. We ask these questions to help us work out the total amount of steroids you have used over time for the research study.

So that you know which of your medications we are interested in, we have provided document titled ‘List of Steroids’ to go with this diary. You will probably be used to hearing these medications being called steroids, but other names you might have heard are ‘glucocorticoids’ and ‘corticosteroids’.

If you have any questions about anything in this booklet please contact:

[CONTACT DETAILS]

| Participant ID | S | A | I | R | A |  |  |  |  |
| --- | --- | --- | --- | --- | --- | --- | --- | --- | --- |

Introduction

Please fill in this diary on the same morning that you collect your saliva sample. You should collect the sample before starting this diary. Remember, you should collect your saliva sample 15 to 30 minutes after you wake up.

It is possible that not all parts of this diary will apply to you – if this is the case you can skip that part.

A tick box is indicated by the symbol 🔾

Part 1 – Collecting your saliva sample

The amount of cortisol in your saliva can be affected by many things, including the time of day, which is why we asked you to follow certain rules when you collected your saliva sample(s). The reason we ask the questions in this section is to help us be sure the results from your sample are a good reflection of your normal cortisol levels. Try to be as accurate as you can when answering, but don’t worry if you aren’t exactly sure when you went to sleep!

1. What is today’s date?

| D | D |  | M | M |  | Y | Y | Y | Y |
| --- | --- | --- | --- | --- | --- | --- | --- | --- | --- |
|  |  | / |  |  | / |  |  |  |  |

1. What time did you wake up this morning?

| H | H |  | M | M |
| --- | --- | --- | --- | --- |
|  |  | : |  |  |

Part 1 continued

1. Roughly what time did you go to sleep last night?

| H | H |  | M | M |
| --- | --- | --- | --- | --- |
|  |  | : |  |  |

1. How did you wake up this morning?

🔾 I woke up naturally

🔾 I was woken up (for example, by an alarm clock)

1. What time did you collect your saliva sample? (for those who collected two samples, tell us only about the first, using kit number 1)

| H | H |  | M | M |
| --- | --- | --- | --- | --- |
|  |  | : |  |  |

1. In the time between waking and collecting your saliva sample(s) did you avoid:

| eating | 🔾 No | 🔾 Yes |  |
| --- | --- | --- | --- |
| brushing your teeth | 🔾 No | 🔾 Yes |  |
| smoking | 🔾 No | 🔾 Yes |  |
| drinking anything but water | 🔾 No | 🔾 Yes |  |

1. Between waking and collecting your saliva sample(s) did you take any steroid medication? (see the List of Steroids if unsure)

| No 🔾 | Yes 🔾 |
| --- | --- |


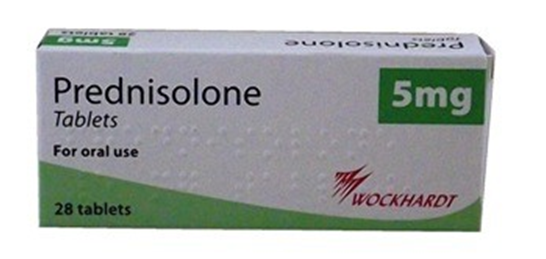


Strength

Name

Part 2 – Have you taken any steroids today?

In this part we would like you to fill in a diary about any steroid tablets you have taken in the past 24 hours. We are interested in any of the medications listed in the List of Steroids. Please use this list to help you fill in the diary. You may find it helpful to complete this section with a friend or family member.

We would like to know the name and the dose of any steroid tablets you have taken in the past 24 hours. We have split the diary into times of day – please write about any tablets you have taken throughout the last 24 hours next to the nearest time of day.

If you are unsure about the name of your medication or the dose you take, the name and strength of your medication will be on its packaging (as shown on the picture below). The dose is the total amount of that medication you take at once. If you prefer, you can write down the strength and the number of tablets you take at once. To help you, we have included some examples of the different ways you could write this information on the first part of the diary.

The strength may be in milligrams (written mg or MG) or micrograms.

| Part 2 continued | | | | |
| --- | --- | --- | --- | --- |
|  |  | Name of drug |  | Dose |
| Examples |  | Prednisolone |  | two 5mg tablets |
|  |  |  |  |  |
|  |  | Prednisolone (gastro |  | 7.5 mg |
|  |  | resistant) |  |  |
|  |  | enteric coated prednisolone |  | one 2.5mg tablet and |
|  |  | tablets |  | one 5mg tablet |
|  |  | Name of Drug |  | Dose |
| Last night (bed time) |  |  |  |  |
|  |  |  |  |  |
|  |  |  |  |  |
|  |  |  |  |  |
|  |  |  |  |  |
|  |  |  |  |  |
|  |  | Name of drug |  | Dose |
| Yesterday evening (around meal time) |  |  |  |  |
|  |  |  |  |  |
|  |  |  |  |  |
|  |  |  |  |  |
|  |  |  |  |  |
|  |  |  |  |  |

| Part 2 continued | | | | |
| --- | --- | --- | --- | --- |
|  |  | Name of drug |  | Dose |
| Yesterday afternoon |  |  |  |  |
|  |  |  |  |  |
|  |  |  |  |  |
|  |  |  |  |  |
|  |  |  |  |  |
|  |  |  |  |  |
|  |  | Name of Drug |  | Dose |
|  |  |  |  |  |
| Yesterday midday (around meal time) |  |  |  |  |
|  |  |  |  |  |
|  |  |  |  |  |
|  |  |  |  |  |
|  |  |  |  |  |
|  |  | Name of Drug |  | Dose |
| Yesterday morning |  |  |  |  |
|  |  |  |  |  |
|  |  |  |  |  |
|  |  |  |  |  |
|  |  |  |  |  |
|  |  |  |  |  |

Part 3 – Did you take any steroids last week?

We would like to know whether you have used any steroid tablets in the past week. This is to help us find out how often people are taking steroids prescribed by *any* doctor (including their GP and rheumatologist). You may find it helpful to complete this section with a friend or family member.

Please tell us about any of the steroid tablets that you have used in the past week – have a look at the List of Steroids if you are unsure. The name and the dose should be filled in as explained in Part 2. The frequency means how many times this week have you taken that dose of the medicine. There are some examples below to help you out.

Examples

| Name of drug |  | Dose |  | Frequency |
| --- | --- | --- | --- | --- |
| Prednisolone |  | 7.5 mg |  | every day |
|  |  |  |  |  |
| Prednisolone |  | 2.5 mg |  | twice a day for |
|  |  |  |  | the last 3 days |
| Prednisolone |  | one 5mg tablet |  | once every other |
|  |  |  |  | day |

Part 3 continued

Have you used any of the medications from the List of Steroids in the past week?

🔾 No

You can go straight to Part 5

🔾 Yes

For those steroids you have used in the past week, please give the following details:

| Name of drug |  | Dose |  | Frequency |
| --- | --- | --- | --- | --- |
|  |  |  |  |  |
|  |  |  |  |  |
|  |  |  |  |  |
|  |  |  |  |  |
|  |  |  |  |  |
|  |  |  |  |  |
|  |  |  |  |  |
|  |  |  |  |  |
|  |  |  |  |  |
|  |  |  |  |  |
|  |  |  |  |  |
|  |  |  |  |  |

Part 4 – Do you ever not take your steroids?

If you answered ‘No’ in Part 3 please skip this section and move onto Part 5.

The following questions ask about the way you use your steroids. We ask these as we know that sometimes people find it difficult to take all their medications all of the time. For us to be sure of our findings we need an accurate idea of the amount of steroids people take. Please try to give truthful answers - this information will not be shared with your doctor or any other healthcare professional.

Think about the steroids you have taken this week and answer the following:

*[SECTION NOT USED]*

Part 5 – Have you had any steroid injections?

In this section, please tell us about any steroid injections you have been given in the past month.

Have you been given any steroid injections in the past month?

- No
- Yes
- Don’t know
- If yes, when were you given the injection? (Consider only the most recent if you had more than one).

| D | D |  | M | M |  | Y | Y | Y | Y |
| --- | --- | --- | --- | --- | --- | --- | --- | --- | --- |
|  |  | / |  |  | / |  |  |  |  |

- What type of injection was it?

| 🔾 Into a joint | 🔾 Into a muscle | 🔾Into a vein |
| --- | --- | --- |

Help:

Injections into muscles are usually into an arm or buttock.

Injections into veins will only ever be given to people when they are in hospital.

If you are not sure whether you were injected into a muscle or a vein, consider the points above and take a look at the diagram to the left.


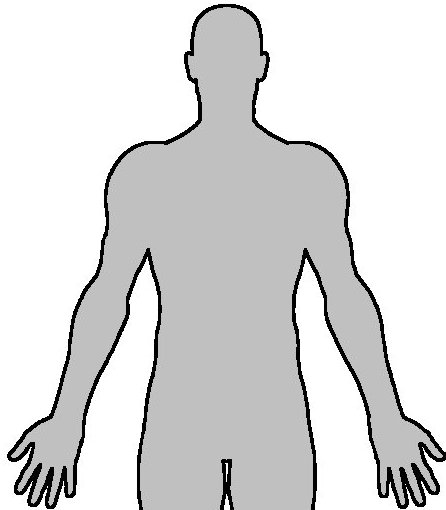


Muscle

Vein

Part 6 – When did you last use steroids?

This final section is for us to make sure we haven’t missed anything, in case you haven’t used any steroids in the past month. You may find it helpful to complete this section with a friend or family member.

We would like to know, to the nearest month, when you last used steroids. Have a look at the List if you are not sure whether your medication was a steroid. Don’t worry if you cannot remember exactly – just try to be as accurate as you can.

1. When was the last time you took a steroid tablet?

| M | M |  | Y | Y | Y | Y |
| --- | --- | --- | --- | --- | --- | --- |
|  |  | / |  |  |  |  |

1. When was the last time you were given a steroid injection?

| M | M |  | Y | Y | Y | Y |
| --- | --- | --- | --- | --- | --- | --- |
|  |  | / |  |  |  |  |

Thank you for completing this diary: your contribution is much appreciated.

Please now return this diary in the envelope provided to:

[ADDRESS]

List of Steroids

This document has been designed to help you fill in the diary.

The steroids that we are interested in are a particular kind of drug that can be called ‘corticosteroids’ or ‘glucocorticoids’. These are the steroids that reduce pain and swelling. On the following pages is a list of the glucocorticoid tablets that are available in the UK. The steroids normally used for rheumatoid arthritis are prednisolone and prednisone; however we would like to know if you have used any of the drugs in the list as they could all affect your level of cortisol.

Help using this list:

Medicines can be referred to by two names: a ‘generic’ name and a brand name. The brand name is chosen by the company that makes it and several companies may make the same generic medicine. To help you identify your medications we have listed the generic names and some of the common brand names.

The generic name will always be on the packaging of your medication. The strength of your medication will also always be on the packaging. The strength may be written in milligrams (mg or MG) or micrograms.

We have included pictures of the tablets to help you identify your medications. In some cases your tablets may appear different to those shown. If you are unsure, match the generic name and strength written on your packaging to those in the list.

Prednisolone

Prednisone 1 mg

Modified release

(Lodotra®)

Prednisone 2 mg

Modified release

(Lodotra®)

Prednisone 5 mg

Modified release

(Lodotra®)

Prednisone

Prednisolone 1 mg


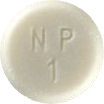


Prednisolone 5 mg


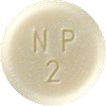


Prednisolone 25 mg


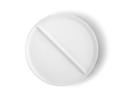

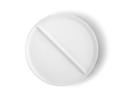

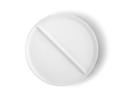


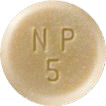


Prednisolone 2.5 mg

Enteric coated / Gastro-Resistant

(Deltacortril®)


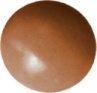


Hydrocortisone 5 mg

Modified Release

(Plenadren®)

Hydrocortisone 20 mg

Modified Release

(Plenadren®)

Hydrocortisone

Hydrocortisone 10 mg

Prednisolone 5 mg

Enteric coated / Gastro-Resistant

(Deltacortril®)


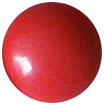

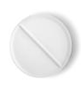

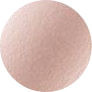

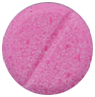


Prednisolone 5 mg

Soluble tablets


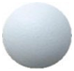


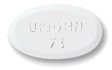


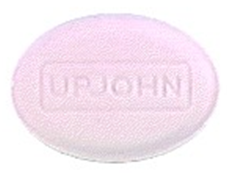

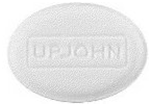

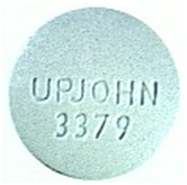

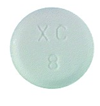

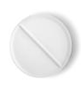


Dexamethasone 2 mg

Dexamethasone

Dexamethasone 2mg / 5ml

Oral Solution

Dexamethasone 500 micrograms

Methylprednisolone 100 mg

(Medrone®)

Methylprednisolone 16 mg

(Medrone®)

Methylprednisolone 4 mg

(Medrone®)

Methylprednisolone

Methylprednisolone 2 mg

(Medrone®)

Deflazacort 6mg

(Calcort®)


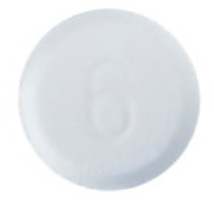


Deflazacort

Betamethasone 500 micrograms

Soluble tablets

(Betnesol®)


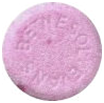


Betamethasone


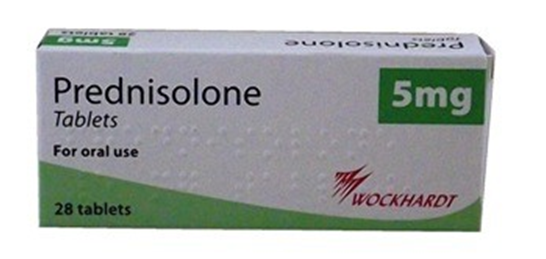


Strength

Name
