# Supplementary material for: Exposure measurement error when assessing current glucocorticoid use using UK primary care electronic prescription data
Source: Pharmacoepidemiol Drug Saf. 2018 Sep 28;28(2):179–86. doi: 10.1002/pds.4649 (PMC6492099; doi:10.1002/pds.4649)
Supplement: Supplementary file 2 — Data S2: Supporting information [file PDS-28-179-s002.docx]

**Supplementary File 2 Overview of data preparation steps used to define current use of oral glucocorticoids**

This analysis used a modified version of an algorithm, written in Stata, which cleans and processes CPRD prescription data to produce a matrix of drug exposure over time. The original algorithm is described below, and the modified version is available to download from Zenodo.com (doi 10.5281/zenodo.1243272).

Implausible values for number of drug units per day were based on maximum values recommended by the BNF plus 10%. Maximum quantity was set as the average number of drug units per day per drug substance multiplied by 6 months.

***The following text and figure is reproduced from:***

Pye SR, Sheppard T, Joseph RM, et al. Assumptions made when preparing drug exposure data for analysis have an impact on results: An unreported step in pharmacoepidemiology studies. Pharmacoepidemiol Drug Saf. 2018;1–8. <https://doi.org/10.1002/pds.4440>

*Available under a creative commons licence (CC BY 4.0* [*https://creativecommons.org/licenses/by/4.0/*](https://creativecommons.org/licenses/by/4.0/)*)*

**Overview of the drug prep algorithm**

In CPRD, individual prescriptions have a date of issue but no end date. This algorithm determines the start and stop date of individual prescriptions. Initially, the issue date is taken to be the start date. An end date is then determined by estimating the duration of each prescription based on multiple sources of information within the raw CPRD data. The algorithm takes into account the following sources of duration information:

- The prescription duration as entered by the prescriber (numdays)
- A duration variable derived from free text notes written by the prescriber (dose_duration)
- The quantity (qty) of drug units (e.g. tablets) in a prescription divided by the daily dose (ndd). The daily dose variable is the number of drug units to be taken per day and is also derived from the free text notes written by the prescriber.

Once each prescription is assigned a start and end date, longitudinal exposure history is assessed. This assessment is made at the product level. Consecutive prescriptions may overlap. The algorithm includes an option to move the start dates of consecutive prescriptions so they no longer overlap, assuming that the patient completes one course before beginning the next. Finally, there may be small gaps between consecutive prescriptions. It is possible that these gaps are artefacts resulting from previous data cleaning steps rather than true breaks in drug exposure. The algorithm therefore includes options to “fill in” these gaps, reclassifying them as exposed time.

**Figure - The original drug exposure preparation algorithm**


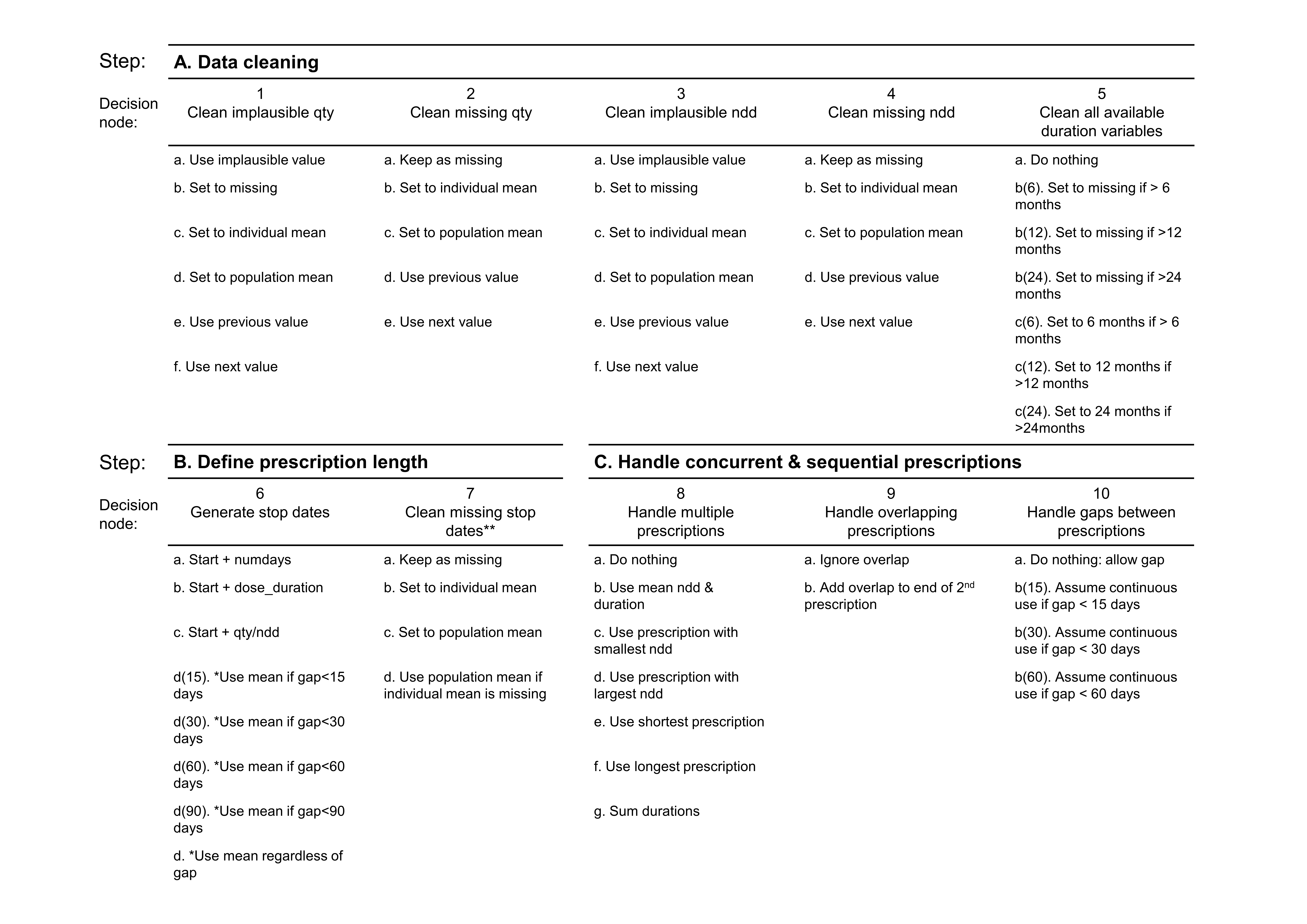


qty = total quantity entered by GP for the prescribed product; ndd = derived numeric daily dose; numdays = number of treatment days; dose_duration = derived duration of prescription. All options that produce a missing value stay coded as missing unless otherwise stated. *For options 6d: If only one stop available, use it; if 2 available and equal, use that date; if 2 available and unequal (but within x days), use mean; if 3 available and unequal, use mean of closest 2 if within x days. **Records with missing stop dates after step 7 are dropped.

The modified algorithm used in the current paper includes some additional options. For purposes of replication, the following options were used:

1. Make cleaning decisions at level of specific drug product
2. Set implausible quantity to missing
3. Set missing quantity to median value for the study population for that drug
4. Set implausible numeric daily dose to missing
5. Set missing numeric daily dose to median value for the study population for that drug
6. Set durations to missing if greater than 6 months
7. Use available duration variables in specified order (derived from free text, average gap between refills, quantity/daily dose, GP reported).
8. Set missing stop dates to median for that individual, otherwise median for the study population
9. Multiple prescriptions on same day: do nothing
10. When prescriptions overlap, move the later prescription to avoid the overlap
11. Assume continuous exposure if gaps between consecutive prescriptions are less than 15 days.
