# Supplementary material for: Exposure measurement error when assessing current glucocorticoid use using UK primary care electronic prescription data
Source: Pharmacoepidemiol Drug Saf. 2018 Sep 28;28(2):179–86. doi: 10.1002/pds.4649 (PMC6492099; doi:10.1002/pds.4649)
Supplement: Supplementary file 3 — Data S3: Supporting information [file PDS-28-179-s003.docx]

**Supplementary File 3 Stata code to generate dataset for hypothetical case-control study**

Description:

Create 100,000 observations

Define true exposure: set exposure to 50%

Define observed exposure: classify 15.8% of exposed as unexposed, classify 12.5% of unexposed as exposed

Define outcome: classify specified proportions of those **truly exposed** and **truly unexposed** as having the outcome

Calculate relative risks for true exposure and observed exposure

Repeat for each of the following true relative risks: 1.5, 4, 9

/***************************************************

* Generates dataset with specified RR and with the

* level of misclassification found in the study,

* i.e. sensitivity 84.2% and specificity 87.5%

***************************************************/

clear

set more off

** set prevalence in unexposed, and

** prevalence in unexposed

/*******************

* NOTES

* To complete table use following:

* RRs

* 1:1.5 12%:18% or 24%:36%

* 1:4 6%:24% or 12%:48%

* 1:9 3%:27% or 6%:54%

*******************/

local unexprevs 0.12 0.24 0.06 0.12 0.03 0.06

local rrs 1.5 1.5 4 4 9 9

foreach observation in 100000 {

foreach num of numlist 1/6 {

drop _all

local unexprev: word `num' of `unexprevs'

local rr: word `num' of `rrs'

** Create dataset

set obs `observation'

** Set TRUE exposure to be 50%

gen trueexp = (_n > (_N/2))

** Set ESTIMATED exposure using the sensitivity and

** specificity to alter the percentage correctly and

** incorrectly classified

bys trueexp: gen obsno = _n

gen perc = obsno / (_N/2)

gen estexp = (round(perc,0.000001)<=0.842) if trueexp == 1

replace estexp = (round(perc,0.000001)<=0.125) if trueexp == 0

tab estexp trueexp if `num'==1

** Generate outcome variable: prevalence is conditional on true exposure

** but independent of estimated exposure

// drop obsno perc

sort trueexp estexp

by trueexp estexp: gen outcome=(_n / _N <= `unexprev') if trueexp==0

by trueexp estexp: replace outcome=(_n / _N <= (`unexprev'*`rr')) if trueexp==1

** Calculate relative risks

di "~~~~~~~~~~~~~~~~~~~~~~~~~~~~~~~~~~~~"

di as text "Overall prevalence: " `unexprev'+`unexprev'*`rr'

qui cs outcome trueexp

di as text "True relative risk: " round(r(rr),0.01)

qui cs outcome estexp

di as text "Estimated relative risk: " round(r(rr),0.01)

di "~~~~~~~~~~~~~~~~~~~~~~~~~~~~~~~~~~~~"

}

}
