# Supplementary material for: Exposure measurement error when assessing current glucocorticoid use using UK primary care electronic prescription data
Source: Pharmacoepidemiol Drug Saf. 2018 Sep 28;28(2):179–86. doi: 10.1002/pds.4649 (PMC6492099; doi:10.1002/pds.4649)
Supplement: Supplementary file 4 — Data S4: Supporting information [file PDS-28-179-s004.docx]

**Supplementary File 4 Flow of participants through the study**

Reproduced from: Joseph RM, Soames J, Wright M, Sultana K, van Staa TP, Dixon WG. Supplementing electronic health records through sample collection and patient diaries: A study set within a primary care research database. Pharmacoepidemiol Drug Saf. 2017;1–4. https://doi.org/10.1002/pds.4323

This article is available under the terms of the Creative Commons Attribution License (CC BY) (which may be updated from time to time) and permits use, distribution and reproduction in any medium, provided that the contribution is properly cited.

Potentially eligible based on EHR:

**3974**

Potentially eligible based on EHR:

**252**

Practice declined

Judged suitable:

**581**

Invited:

**526**

Positive response:

**125**

Recruited:

**117**

Completed screening:

**77**

Completed mail-out:

**66**

Practice did not respond

-969

-71

-2146

-104

Screened:

**859**

Judged unsuitable

-278

Practice did not mail-out

-55

-11

Negative response:

No response:

-11

-391

Unresolved problem with consent form

-3

Responded after deadline

-3

Lost to follow-up

-21

Withdrew

-8

Did not return diary

-2

**PRACTICES**

**PATIENTS**

Completed study:

**86**

No contact details

-2

Practice recruitment

Screening and mail-out

Participant recruitment

Completing the study

### Table S4.1 Patient characteristics by stage of recruitment

*n number; IQR inter-quartile range, DMARDs disease-modifying anti-rheumatic drugs; GP general practice; CPRD Clinical Practice Research Datalink*

*in the year prior to 30/11/2015

| Sub-population | Number of participants | Female, n (%) | Age (years), median (IQR) | Townsend score quintile, median (IQR) | DMARDs - ever*, n (%) | GP visits* (n), median (IQR) |
| --- | --- | --- | --- | --- | --- | --- |
| All eligible patients within CPRD | 3718 | 2640 (71%) | 68 (58-77) | 3 (2-4) | 2348 (63.2%) | 2 (0-4) |
| Practice agreed to mail-out | 761 | 535 (70.3%) | 69 (59-77) | 3 (2-4) | 566 (74.4%) | 3 (2-6) |
| Patient recruited to study | 117 | 84 (71.8%) | 69 (60-74) | 3 (2-3) | 92 (78.6%) | 4 (2-6) |
| Participant returned sample and diary | 86 | 60 (69.8%) | 68.5 (60-74) | 2 (2-3) | 67 (77.9%) | 3 (1-6) |
